# Clinical outcome of patients with differentiated thyroid cancer and raised antithyroglobulin antibody levels: a retrospective study

**DOI:** 10.1186/s13044-021-00099-w

**Published:** 2021-04-15

**Authors:** Manish Ora, Aftab Hasan Nazar, Prabhakar Mishra, Sukanta Barai, Amitabh Arya, Prasanta Kumar Pradhan, Sanjay Gambhir

**Affiliations:** 1 Department of Nuclear Medicine, SGPGIMS, Lucknow, India; 2 Department of Biostatistics and Health Informatics, SGPGIMS, Lucknow, India

**Keywords:** Differentiated thyroid cancer, Serum antithyroglobulin antibody, Radioiodine therapy, Recurrence

## Abstract

**Background:**

Thyroglobulin (Tg) is a specific tumor marker for differentiated thyroid cancer (DTC). However, in the presence of an antithyroglobulin antibody (TgAb), it becomes unreliable. The purpose of the study was to assess the long-term outcome of DTC patients with raised TgAb.

**Method:**

In a retrospective study, we included patients with DTC who had raised TgAb following total thyroidectomy. We excluded patients with persistently raised Tg (≥ 1 ng/ml) or radioiodine avid disease. Serial TgAb levels, excellent response (ER), incomplete response (IR), and anatomical recurrence were evaluated.

**Results:**

A total of seventy-six patients were included in the study. Patients with IR had higher baseline TgAb (1071.27 ± 1216.17 vs. 99.61 ± 91.29 IU/ml, *p* < 0.001) and central compartment lymph node metastases (70.8% vs. 46.4%, *p* = 0.035) in comparison to those in the ER group. In the first follow-up, 64 (84.2%) patients had a stable or fall in the TgAb (0 to − 98.3%). Sixty-eight patients received high-dose radioiodine therapy (RIT). Out of these, 59 (86.5%) had transient, and 51 (75%) had a long-term fall in TgAb. After a follow-up period of 58.74 ± 26.26 months, 63.2% (48 out of 76) patients had IR. Nine (11.8%) patients had a rising TgAb level (3.7–170.9%) from baseline. Eleven patients underwent 18F-FDG PET/CT, and five of them demonstrated metabolically active recurrent disease. Three patients underwent cervical lymph nodes dissection. None of the patients died during the follow-up period.

**Conclusion:**

High post-operative TgAb levels and central compartment lymph nodal metastases are risk factors for IR. RIT leads to a significant fall in the TgAb in these patients. The low level of raised TgAb is associated with an excellent outcome. Patients with recurrences had very high baseline TgAb > 1000 IU/ml. Raised TgAb was associated with good clinical outcomes and not associated with increased mortality.

## Introduction

Differentiated thyroid cancer (DTC) is the most common of the endocrine cancers. It accounts for 3.1% of all malignancies globally, with an age-standardized incidence of 7.4/ 100,000 people. However, because of the excellent 5 and 10 years survival rate in comparison to other solid malignancies, it is the fifth most prevalent cancer in the world [1].

Treatment of DTC consists of total thyroidectomy or lobectomy. Surgery is followed by evaluation of post-operative disease status. It consists of a diagnostic whole-body radioiodine scan (WBS), serum thyroglobulin (Tg), and antithyroglobulin antibody (TgAb) assay [2]. The need for radioiodine therapy (RIT) depends on the patient’s disease stage and risk factors [2]. Serial Tg and TgAb assays and neck ultrasonography (USG) with or without WBS are currently the mainstays of post-operative surveillance in patients with DTC.

Tg is the specific tumor marker of the DTC and is a critical investigation to identify patients with residual or recurrent disease. Tg is only produced by thyroid tissue or well-differentiated thyroid cancer tissue, so serum Tg level helps detect recurrent or residual disease in DTC patients who have undergone total thyroidectomy and RIT. However, in the presence of TgAb, Tg measurement becomes unreliable and may fail to identify patients with significant residual or recurrent tumors [2, 3]. The variability in TgAb assays may also result in falsely negative TgAb levels [3]. It is associated with a misleadingly low serum Tg due to the presence of the antibodies that the assay could not detect [3]. Both Tg and TgAb assays may be affected by heterophilic antibodies [4, 5].

Nearly 20% of DTC patients show circulating TgAb [3]. It is commoner in patients with autoimmune Hashimoto’s thyroiditis (HT). The presence of TgAb should be suspected when the pathology indicates the background HT. [6] Some studies have suggested an association between autoimmune thyroid disease and papillary thyroid cancer. However, the prognostic significance of this finding is not well understood [3, 7–9]. Destruction of normal follicular thyrocytes or neoplastic cells may reduce or even eradicate TgAb by removing the antigenic stimulus [10]. However, persistent or increasing serum TgAb level in DTC patients during their follow-up is a recurrent/persistent disease marker. Several authors have reported the association between DTC aggressiveness and TgAb with discordant results [11–16]. However, the long-term clinical outcome and clinical significance of the raised TgAb are not very well studied.

This retrospective single-center study aims to evaluate the DTC patient with raised serum TgAb titers after surgery. We aim to assess the changes in the TgAb levels after radioiodine ablation and the patients’ long-term clinical outcome.

## Material and methods

### Patients selection

**Fig. 1:**
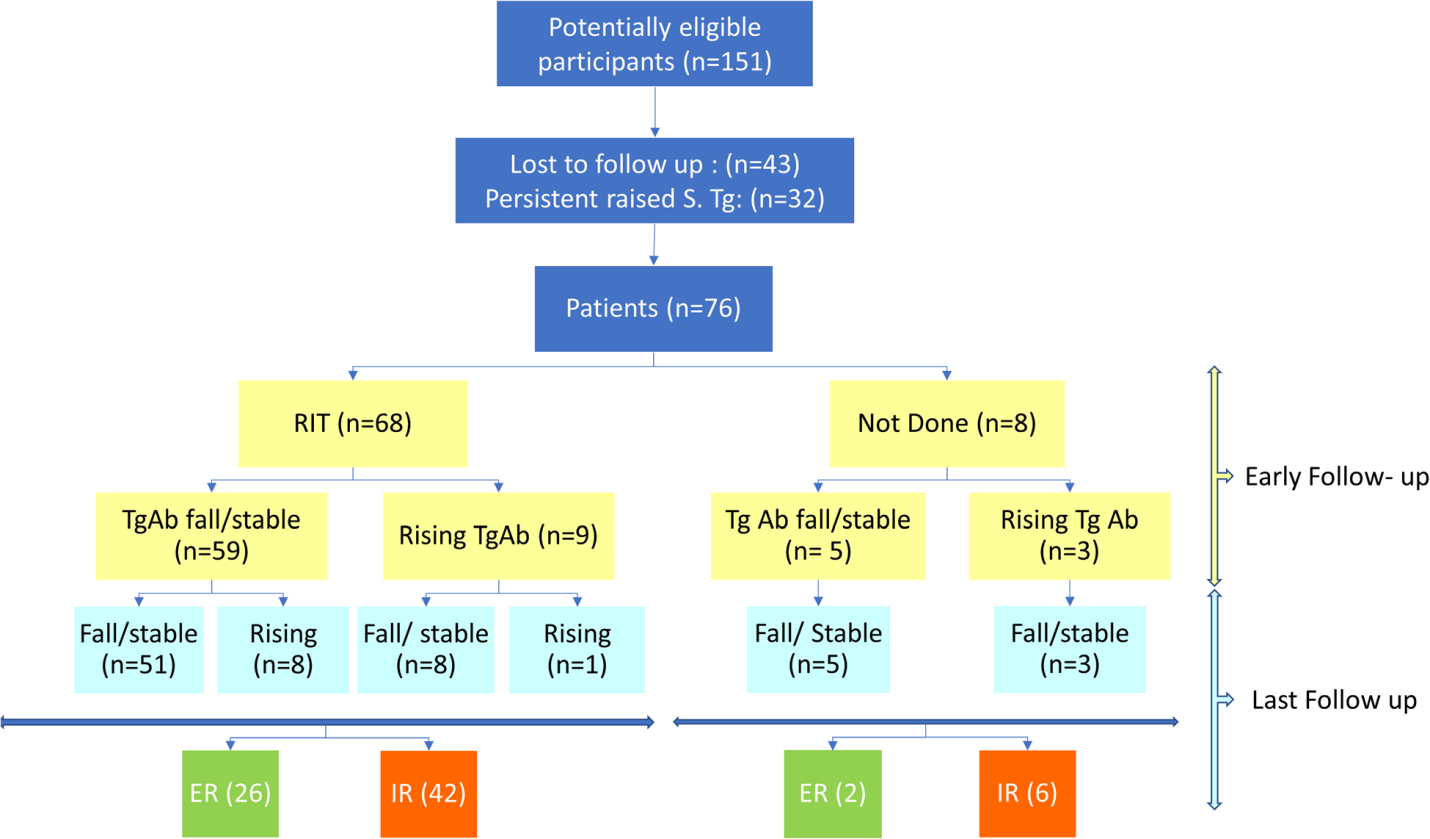
Flow Chart shows the recruitment of the study subjects, early and final response in the patients with their outcomes. The number in parenthesis shows the number of patients. (RIT: High dose Radioiodine Therapy, ER: excellent response, IR incomplete response)

This retrospective study included DTC patients treated in the Nuclear Medicine department between January 2005 and December 2018. All patients underwent total thyroidectomy at our institution or a regional hospital. Patients underwent central compartment neck dissection (CCLND) in advanced primary tumors, biopsy-proven lymph node metastases, or suspicious neck USG findings preoperatively. All patients with biopsy-proven metastatic lateral cervical lymphadenopathy underwent lateral neck lymph node dissection (LLND). The patients were included in the study after considering the following inclusion and exclusion criteria (Fig. 1).

#### Inclusion criteria

1. A raised serum TgAb titer at the initial post-operative assay after total thyroidectomy and before WBS or RIT
2. Stimulated serum Tg level less than 1 ng/dl in first follow up (after 6 months)
3. A negative WBS scan in the first follow-up
4. Adequate clinical follow-up data for at least 12 months is available.

#### Exclusion criteria

1. Patients with both elevated Tg (≥1 ng/dl) and TgAb in follow-up
2. Patients with anaplastic carcinoma or with a poorly differentiated variant of papillary thyroid carcinoma (such as the insular, tall cell variant)
3. Patients who were lost to follow up
4. Patients have positive WBS in follow-up (persistent iodine avid local or distant metastatic disease).

### Radioiodine ablation

All patients were prepared by withdrawing thyroid hormone replacement for 1 month. Radioiodine was given for WBS to all patients with TSH levels above 50 mIU/L. Stimulated serum Tg (Immunoradiometric assays with functional sensitivities up to 0.2 ng/mL), TgAb (radioimmunoassay RIA) were measured in all the patients. Radioiodine therapy (RIT) was administered to all patients with positive WBS. After that, all patients received hormonal suppression therapy to keep a TSH level according to the risk category and response to therapy during the follow-up according to the American Thyroid Association (ATA) guidelines [2].

### Evaluation of treatment response and clinical management during follow-up

All patients were evaluated 6 months after the RIT for early response assessment. The initial response to therapy was assessed by serum Tg and TgAb measurements and WBS. After the first follow-up, patients were reassessed with stimulated/suppressed serial Tg and TgAb measurements, neck USG with or without WBS every 6–12 months. In patients with very high or rising TgAb, ^18^F- FDG PET-CT was done. Ultrasound-guided fine-needle aspiration cytology (FNAC) was performed in all patients with suspicious neck lesions. Patients with recurrent disease are referred for surgery.

### Definition of the outcome

1. A stimulated Tg < 1 ng/ml, absence of TgAb, no uptake on WBS in follow-up was defined as an excellent response (ER).
2. A negative WBS stimulated serum Tg < 1 ng/mL with persistent/declining TgAbwas considered an indeterminate response.
3. A negative WBS, stimulated Tg < 1 ng/mL with increasing TgAbwas considered as a biochemical incomplete response (BIR) [2].

In -follow-up, patients were reclassified as ER if patients with BIR or intermediate response showed a stimulated Tg < 1 ng/ml, undetectable TgAb, and no evidence of suspicious disease on WBS or USG. For this study, patients with rising (BIR) or stable/ declining TgAb (indeterminate response) were considered incomplete response (IR) groups. Any rise in the level of TgAb in the follow-up compared to the baseline was considered as rising TgAb. Falling TgAb was considered if patients had any falls in the level of the TgAb.

### Data collection and analysis

Clinical data included patient demographics, tumor characteristics (histological features, baseline tumor stage), treatments (the type of surgery, RIT), laboratory and imaging finding (serial serum Tg and TgAb levels, and ^18^F -FDG PET-CT), and outcome (disease status and cause of death if applicable). Patients were stratified using the eighth edition of the American Joint Committee on Cancer/International Union against Cancer (AJCC/UICC) staging system [17].

### Statistical analysis

The normality of the continuous variables was established. A variable was considered normally distributed when the Z value of the skewness was within ±3.29. The normality of the continuous variable was expressed as means ± standard deviation while categorical variables as frequencies (%). Independent samples t-test was used to compare the means between the two groups. Chi-square tests (or Fisher exact test) were performed to test the association between two categorical variables. The receiver operating characteristics (ROC) curve was used to calculate the area under the curve (AUC) and appropriate cutoff value with corresponding sensitivity and specificity for the detection of IR by TgAb levels. Data analysis was done on the Statistical Package for Social Sciences (IBM Corp. Released 2015. IBM SPSS Statistics for Windows, Version 23.0. Armonk, NY: IBM Corp).

## Results

### Baseline characteristics

The study included a total of seventy-six patients (female: 63, 82.8%). A baseline summary of patient characteristics, including demography, primary surgery, histopathology details, and biochemical parameters, are presented in Tables 1 and 2. The mean age of presentation was 35.71 ± 14.01 years, with a range of 10–75 years. Nearly three fourth (77.6%) and a half (46.1%) of the patients underwent CCLND and LLND, respectively. The classical variant of papillary carcinoma was the most common (90.8%) histopathological variant, followed by the follicular variant. One-third (32.9%) of the patients had metastasis limited to the central compartment, and another one-third (35.5%) also had lateral neck lymph nodal involvement. Baseline (median, range) serum Tg and TgAb were 0.27 (0.2–121) ng/dl and 166.5 (30–4000) IU/ml, respectively.

**Table 1:** Descriptive characteristics of the study patients (*N* = 76)

Variables	Mean	Median	Percentile (Q1, Q3)
Age (years) at diagnosis	35.71 ± 14.01	34	25, 45
Time between Surgery and RIT	3.64 ± 1.65	3.0	3, 4.8
RIT (mCi)	66.05 ± 41.12	60.0	30,100
Tg Baseline^a^	5.98 ± 16.78	0.27	20, 2.80
Tg after RIT^a^	0.66 ± 1.24	0.20	0.20, 0.79
TgAb (baseline)^b^	713.29 ± 1073.6	166.50	70, 675.3
TgAb after RIT^b^	420.23 ± 830.56	103.0	10,339.5
TgAb (% change, Baseline-early follow up)	−34.29 ± 68.62	−47.57	−78.97, −11.96
TgAb (final)^b^	450.42 ± 1185.20	53.0	10, 245.5
TgAb (% change, Baseline-last follow up)	−52.62 ± 53.16	−73.48	−86.90, −33.40
Follow up Duration (months)	58.74 ± 26.26	55	42.30, 71.8

*RIT* High dose Radiodiodine Therapy, *Tg* Serum Thyroglobulin, *TgAb* Serum Antithyroglobulin antibody

^a^Tg level expressed in ng/dl

^b^TgAb level expressed in IU/ml

**Table 2:** Characteristic of the patient’s groups based on TgAb response (*N* = 76)

Variables	Incomplete response (***N*** = 48, 63.2%)	Excellent response (***N*** = 28, 36.8%)	Total (***N*** = 76)	***P***-value
^a^Age (Years)	37.75 ± 14.96	32.21 ± 11.66	35.71 ± 14.01	0.077
Sex (Female)	40 (83.3)	23 (82.1)	63 (82.9)	0.990
$TgAb (baseline)	1071.27 ± 1216.17	99.61 ± 91.29	713.29 ± 1073.55	**< 0.001**
**Surgery**				
CCLND (Yes)	41 (85.4)	18 (64.3)	59 (77.6)	**0.033**
LLND (Yes)	27 (56.3)	8 (28.6)	35 (46.1)	**0.020**
**Histopathology**				
HPE CCLND (Yes)	34 (70.8)	13 (46.4)	47 (61.8)	**0.035**
HPLND (Yes)	20 (41.7)	6 (21.4)	26 (34.2)	0.073
Pathology				
PCT	43 (89.6)	26 (92.8)	69 (90.8)	0.991
PCT FV	3 (6.3)	2 (7.1)	5 (6.6)	
FCT	1 (2.1)	1 (3.6)	2 (2.6)	
T stage				
1	3 (6.3)	6 (21.4)	9 (11.8)	0.228
2	9 (18.8)	4 (14.3)	13 (17.1)	
3	7 (14.6)	5 (17.9)	12 (15.8)	
X	29 (60.4)	13 (46.4)	42 (55.3)	
N Stage				
0	7 (14.6)	13 (46.4)	20 (26.3)	**0.010**
N1a	16 (33.3)	9 (32.1)	25 (32.9)	
N1b	22 (45.8)	5 (17.9)	27 (35.5)	
X	3 (6.3)	1 (3.6)	4 (5.3)	
**AJCC staging**				
1	39 (81.3)	27 (96.4)	66 (86.8)	**0.0345**
2	7 (14.6)	1 (3.6)	8 (10.5)	
3	2 (4.1)	0 (0)	1 (2.7)	
**WBS findings**				
Positive	42 (87.5)	26 (92.8)	68 (89.5)	0.703
Remnant only (Yes)	38 (79.1)	21 (75.0)	59 (77.6)	0.519
LN on WBS	10 (20.8)	7 (25.0)	17 (22.4)	0.674
LN Metastases ^b^(Yes)	39 (81.5)	17 (60.7)	56 (73.7)	**0.005**

*LN* Lymph node, *TgAb* antithyroglobulin antibody, *CCLND* central compartment lymph node, *LLND* lateral compartment lymph node, *HPE* histopathology, *PCT* papillary carcinoma thyroid, *FV* follicular variant, *FCT* follicular carcinoma thyroid, *AJCC* American joint committee for cancer, *WBS* whole-body radioiodine scan

*p* < 0.05 is significant and are shown in bold fonts, All parenthesis are percentages

^a^Independent samples t-test used $ Mann Whitney U test. Chi-square test / Fisher exact test was used

^b^LN metastases are either lymph node metastases on HPE or LN on WBS

### Radioiodine therapy and early response assessment (Fig. 1)

**Fig. 2:**
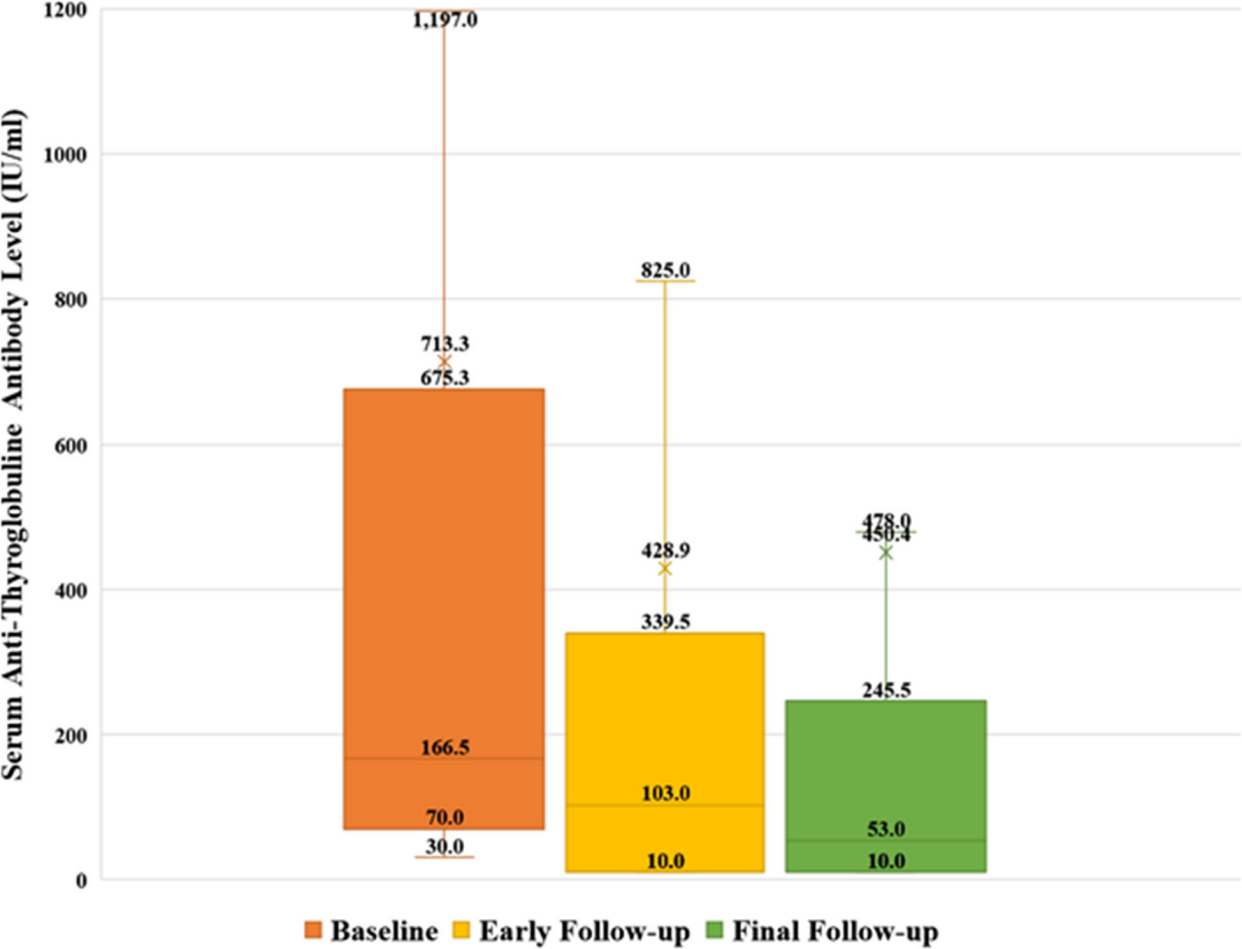
The box plot shows a trend in Antithyroglobulin antibody levels in the baseline, early, and final follow-up. Note that all the outlier data are not shown

The WBS was positive in 68 (89.5%) of the patients. Fifty-three patients only had the remnant. Thirteen patients had involvement of cervical lymph nodes on WBS. Of these 13 patients, three also had mediastinal lymph node involvement, and one had lung metastasis.. These patients received 66.05 ± 41.12 mCi of RIT {1.11–5.55 GBq (30–150 mCi of 131I)}. Eight patients with negative WBS did not receive RIT. In the first follow-up after 6 months, 64 (84.2%) patients had a stable or fall in the TgAb, ranged from 0 to − 98.3%. Of these, twenty (26.3%) patients had ER. However, at the first follow-up, twelve patients (15.8%) had a rising TgAb level (1.95–334%). (Figs. 1 and 2).

### Long term follow up and change in the TgAb level (Figs. 1, 2 and 3)

Patients were in the long-term follow-up (58.74 ± 26.26 months). TgAb became undetectable (ER) in a total of 28 patients. The rest of the 48 (63.2%) patients were in IR, with TgAb level ranges between 22 and 8000 IU/ml. Out of these 48 patients, 21 patients had a TgAb titer less than 100 IU/mL. Out of 59 patients who had fallen TgAb after RIT, 51 patients had persistent falling levels in the follow-up. Eight patients had a rise (3.7–334.4%) in the TgAb after RIT. None of the patients in the ER group showed reappearance of the TgAb. At the time of the final analysis, only nine (11.8%) patients had a rising TgAb level (3.7–170.9% from the baseline). Out of these, only two had the recurrent disease. Total ten patients had moderately raised TgAb (500–1000 IU/ml). None of them had recurrent disease.

**Fig. 3:**
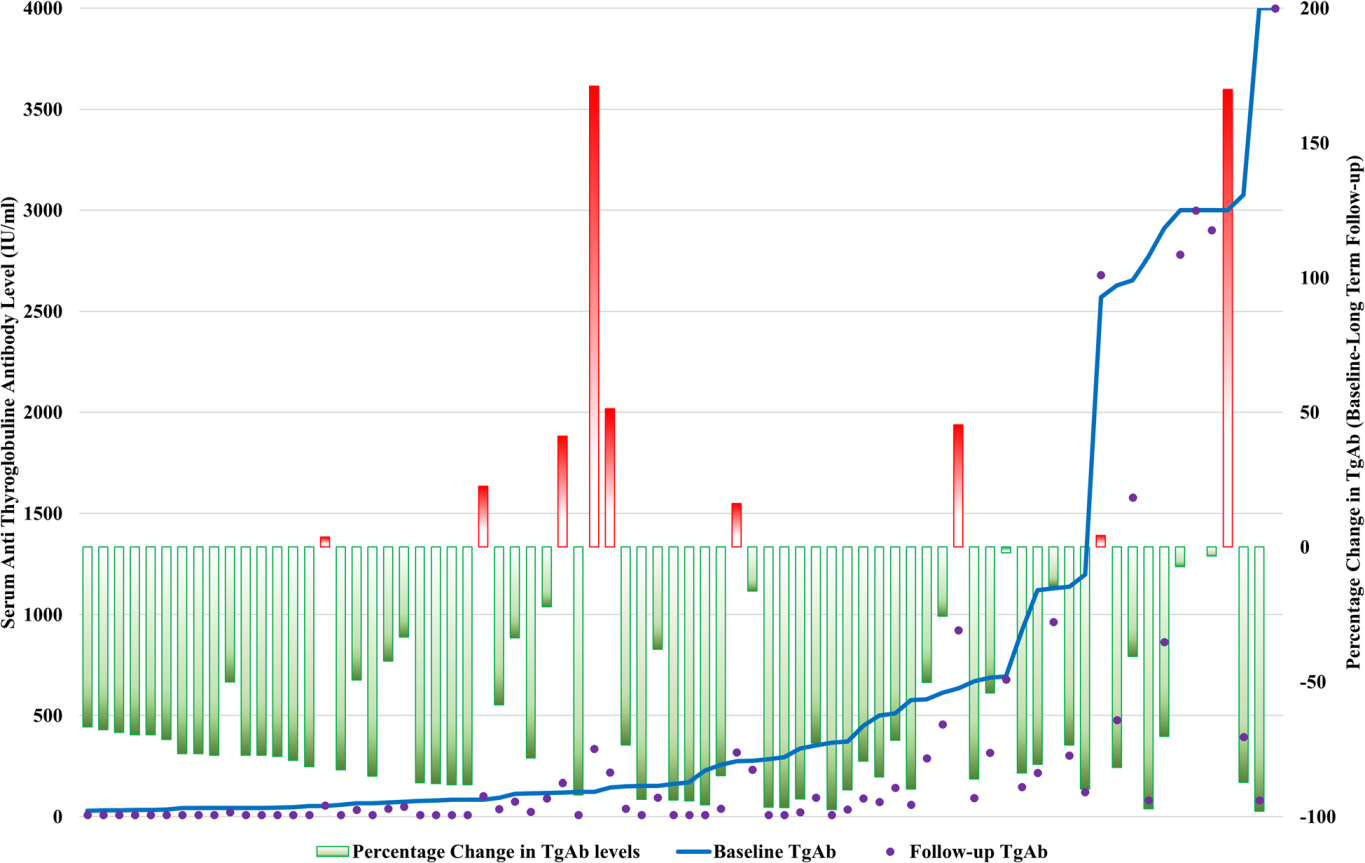
Patients are shown as ascending baseline serum TgAb level (Primary Y-axis). The secondary Y-axis presents percentage change in the TgAb level from baseline to last follow-up (green bars: falling TgAb, Red Bars: rising TgAb). TgAb: Antithyroglobulin antibody

### Structural recurrence and role of 18F FDG PET-CT

A total of 11 (14.47%) patients underwent 18F FDG PET-CT (diagnostic CT along with intravenous contrast agent) for the significantly raised (510–4000 IU/ml) stable or rising TgAb. All the patients with the TgAb less than 1000 IU/ml had no lesions on the PET-CT. Out of these, five patients demonstrated metabolically active lesions. Thyroid bed recurrence, lymph node involvement, and metastatic lung nodules were noted in 1, 5, and 2 patients. The standardized uptake value (SUVmax) of cervical lymph nodes was 3.8–8.8. All patients underwent USG guided FNAC and showed results suggestive of disease recurrence. Three patients underwent lymph nodes dissection for the recurrent disease. HPE was suggestive of lymph nodal metastasis in these patients. None of the patients died during follow-up.

### Predictors of incomplete response after surgery: Univariate analysis (Table 2)

In the IR group, the patients were older than patients in the ER group (37.75 ± 14.96 vs. 32.21 ± 11.66, *P* = 0.077), although the difference was not statistically significant. The IR group had higher baseline TgAb levels (1071.27 ± 1216.17 IU/ml vs. 99.61 ± 91.29 IU/ml, *p* < 0.001), CCLND (85.4 vs 64.3%, *p* = 0.033), LLND (56.3% vs 28.6%, *p* = 0.002), and central compartment lymph node metastases (70.8% vs. 46.4%, *p* = 0.035) in comparison to the ER group. No combination of variables was significantly associated with IR or ER on multivariate binary logistic regression analysis.

### Prognostic value of pre-ablative baseline TgAb for incomplete and excellent response

**Fig. 4:**
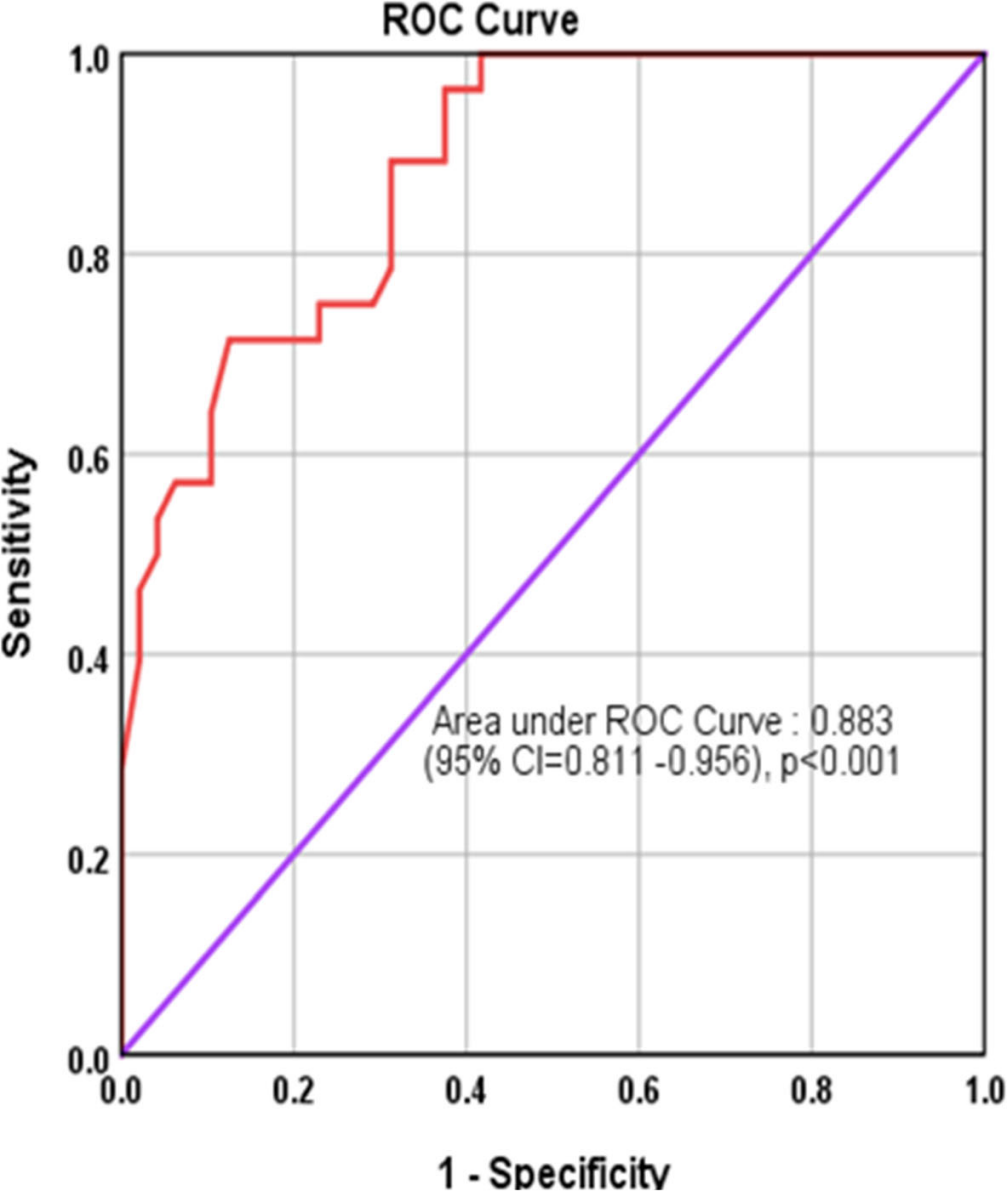
ROC curve showing diagnostic accuracy of the baseline Antoithyroglobulin antibody level to detect the expected fall in TgAb during the follow-up

ROC curve was drawn to find the diagnostic accuracy of the TgAb level to identify the IR and ER. AUC was 88.3% (95% CI = 81.1–95.6, *p* < 0.001) **(**Fig. 4**)**. From all the available cut-off values of TgAb level, three cut-off values i.e. at TgAb≤241.5 IU/ml (Sensitivity = 89.3%, Specificity = 68.7%), TgAb≤158 IU/ml (Sensitivity = 78.6%, Specificity = 69.7%), and TgAb≤89 IU/ml (Sensitivity = 71.4%, Specificity = 87.5%) were considered as the best cut-off value.

## Discussion

The study included a total of seventy-six patients. A large proportion of the patients underwent CCLND and LLND. Lymph nodal metastases were common in our cohort and were noted in 56(73.7%) patients. Baseline (median, range) serum Tg and TgAb were 0.27 (0.2–121) ng/dl and 166.5 (30–4000) IU/ml, respectively. The WBS was positive in 68 (89.5%) of the patients, and they received RIT. After 6 months, 84.2% of patients had a stable or fall in the TgAb range from 0 to − 98.3%, and twenty (26.3%) had ER. After a follow-up (58.74 ± 26.26 months), ER was noted in 28 patients. In the rest of the 48 (63.2%) with IR, the TgAb level ranges from 22 to 8000 IU/ml. Out of 59 patients who had fallen TgAb after RIT, 51 patients had persistent falling levels in the follow-up. A total of 11 patients underwent 18F FDG PET-CT. Five patients demonstrated metabolically active lesions, and three patients underwent cervical lymph nodes dissection. None of the patients died during follow-up. The IR group had higher baseline TgAb levels, CCLND, LLND, and central compartment lymph node metastases. The baseline TgAb ≤241.5 IU/ml has excellent sensitivity (89.3%) for predicting IR.

Long-term follow-up of DTC patients is required to detect recurrence. Serum Tg remains the cornerstone of the investigation. However, persistent TgAb may cause Tg levels to be variable. Approximately 20% of DTC patients and 10% of the general population show TgAb [15, 18]. There may be an early transient decline in TgAb from increased formation and metabolic clearance of Tg-TgAb complexes formed by a rise in Tg with surgery [19]. Destruction of the follicles may lead to an acute increase in Tg antigen and production of TgAb. TgAb may rise or become detectable in response to thyroid surgeries, FNAC, biopsy, or RIT [15, 20–23]. After that, in the absence of a thyroid tissue mass and Tg antigen, TgAb concentrations decline over several months [24, 25]. The median half-life of TgAb after surgery for DTC is 10 weeks [24]. We observed a transient rise in TgAb in comparison to baseline after RIT in 8 patients. All of them had a subsequent fall in the TgAb in the long-term follow-up.

Raised serum TgAb in DTC presents a unique clinical scenario and much concern to the patient and the physician. TgAb interference causes an underestimation of serum Tg when measured by IMA. However, Tg could be either under-or overestimation when measured by RIA. Minimal TgAb concentrations may interfere with Tg without being detected by a few methods. The interference is minimal when Tg (RIA) in the specimen is high and TgAb is low. However, it becomes significant when Tg is low and TgAb is high [3]. Rising TgAb levels after initial treatment are considered as a ‘biochemical incomplete response’ [2, 26]. Several studies have demonstrated an increased risk of recurrence/persistent disease with a new appearance of TgAb or rising titers [14, 24, 27].

After surgery and RIT, the mean TgAb disappearance time is about 3 years [24, 25]. A persistently low and declining TgAb concentration after the initial surgery does not always indicate recurrence [15]. It demonstrates the importance of long-term follow-up in this subset of patients. Our study did not find any structural recurrence in 27 (35.52%) and 51(67.05%) patients with baseline TgAb levels less than 100 and 500 IU/mL, respectively. However, recurrences were seen in 5 (31.25%) patients with TgAb> 1000 IU/mL. Kim et al. noted similar results and found that the recurrence rate was 18 and 1% for serum TgAb > 100 U/mL and TgAb ≤100 U/mL, respectively [15]. We found that the baseline TgAb correlates with the patient’s outcome (ER: 99.61 ± 91.29 and IR: 1071.27 ± 1216.17, *p* = 0.001). Similar observations were seen by a few but not by all previous authors [16, 28, 29].

Our results confirm a favorable long-term outcome in patients with early normalization or significant fall in TgAb titers. Few authors have supported this finding [14–16]. In our study, 59 (86.8%) patients had stable or fell off (0 to − 98.3%) TgAb levels after RIT. Approximately one-third had an ER. Of these 59 patients, 51 (86.4%) patients had a persistently low TgAb level in follow-up. None of them had a rising TgAb or recurrence in follow-up. It may be due to the eradication of functional thyroid cells, benign or malignant, eliminating the antigenic stimulus [3, 25]. .Like our study, a previous study demonstrated that less than 1% of patients with 50–100% decline in TgAb after RIT had a recurrence. However, 37% of patients with rising TgAb and 19% of patients in whom the fall was less than 50% had recurrences [15].

In our study, only five patients (6.5%) had an anatomical recurrence in follow-up, similar to a study by Durante et al. [10]. However, the recurrences were much lower in comparison to other studies (13.6–49%) [14, 15, 30]. This result may be due to different study populations. One of the primary inclusion criteria of our study was a negative follow-up WBS scan. This rules out the possibility of the residual normal or malignant iodine avid thyroid tissue. However, many previous studies did not have it as an inclusion criterion [10, 15, 16, 31]. It raises the possibility of minimal residual tissue in these studies that produced Tg and a persistent TgAb. It could be an associated reason for high structural recurrence in those studies. Despite high but stable TgAb levels, many patients remained asymptomatic with no evidence of structural recurrence and did not require active interventions.

Our study found that the history of lymph node dissection and lateral compartment lymph nodal metastasis were significantly associated with IR (45.8%, *p* = 0.01). Like our observation, Tsushima et al. also showed that N1b lymph nodes metastases and recurrence were common in patients showing < 50% fall or rising TgAb in follow-up [30]. We found that the IR group had higher initial TgAb levels and TNM stages. However, the TNM stage was not a significant factor in multivariable regression analysis.

In a large meta-analysis consisting of 38 studies (10 648 patients), 23.8% of DTC patients had coexisting Hashimoto’s Thyroiditis (HT). It was associated with favorable clinicopathological characteristics, with no extrathyroidal extension (*p* = 0.002), lymph node metastasis (*p* = 0.04), and more prolonged recurrence-free survival (*p* = 0.001) [9]. However, some studies have shown contradicting results [11–13, 15]. We did not evaluate the coexisting HT in our study. We utilized FDG PET-CT to assess these patients as it is considered an excellent method to detect recurrent disease [32].

Our study has several limitations, such as its retrospective design and the low number of patients in individual groups. Longer follow-up is needed as patients may develop recurrence at a later date. All the patients with IR did not undergo FDG PET-CT, as our center has a high FDG PET/CT threshold. We investigated only 12 patients with TgAb > 500 IU/ml. It may be a reason for the lower recurrence in our study. Future research with a large number of subjects is needed.

## Conclusion

This study demonstrated that high post-operative TgAb level and lateral compartment lymph nodal metastases are the risk factor for persistent high TgAb level and incomplete treatment response in long-term follow-up. Radioiodine therapy decreases TgAb levels in a large number of patients. The low level of raised TgAb (< 1000 IU/ml) is associated with an excellent outcome even with a rising TgAb level. Anatomical recurrence is noted at high TgAb levels (> 1000 IU/ml) and involves the neck and thoracic region. Overall, raised TgAb was associated with good clinical outcomes and not associated with increased mortality.

## Data Availability

Available (partial) after adequate permission from the institute.

## Abbreviations

DTC: differentiated thyroid cancer
Tg: Thyroglobulin
TgAb: Antithyroglobulin antibody
RIT: High-dose radioiodine therapy
18F-FDG PET-CT: [18F]Fluoro-2-deoxy-2-d-glucose positron emission tomography- computed tomography
